# TGF-β1 promotes human breast cancer angiogenesis and malignant behavior by regulating endothelial-mesenchymal transition

**DOI:** 10.3389/fonc.2022.1051148

**Published:** 2022-11-16

**Authors:** Zi-Xiong Li, Jie-Xin Chen, Ze-Jun Zheng, Wang-Jing Cai, Xiong-Bin Yang, Yuan-Yuan Huang, Yao Gong, Feng Xu, Yong-Song Chen, Ling Lin

**Affiliations:** ^1^ Department of Rheumatology and Immunology, The First Affiliated Hospital of Shantou University Medical College, Shantou, China; ^2^ Department of Thyroid and Breast Surgery, The First Affiliated Hospital of Shantou University Medical College, Shantou, China; ^3^ Department of Rheumatology, Shantou University Medical College, Shantou, China; ^4^ Department of Respiratory and Critical Care Medicine, The First Affiliated Hospital of Shantou University Medical College, Shantou, China; ^5^ Department of Endocrinology, The First Affiliated Hospital of Shantou University Medical College, Shantou, China

**Keywords:** TGF-β1, EndMT, angiogenesis, breast cancer, BCSLC, dorsal skinfold window chamber

## Abstract

**Background:**

Endothelial-mesenchymal transition (EndMT) is an important process of angiogenesis, which plays a significant role in in tumor invasion and metastasis, while its regulatory mechanisms in breast cancer remain to be fully elucidated. We previously demonstrated that tumor-associated macrophages (TAMs) can induce EndMT in endothelial cells by secreting CCL18 through the activation of the TGF-β and Notch signaling pathways in breast cancer. This study was designed to study the role of EndMT in breast cancer angiogenesis and progression in order to explore the underlying mechanism.

**Methods:**

Immunohistochemistry (IHC) was used to evaluate the expression of microvascular density (MVD) and EndMT markers in breast cancer. TGF-β1 was used to induce EndMT models of differentiated-endothelial breast cancer stem-like cells (BCSLCs). *In vitro* cell migration, proliferation and matrigel tube-formation assays, as well as *in vivo* nude mouse tumor-bearing model and nude mouse dorsal skinfold window chamber (DSWC) model, were utilized to investigate the effects in order to explore the mechanism of EndMT induced by TGF-β1 on breast cancer progression.

**Results:**

In this study, we demonstrated that the EndMT markers were positively associated with MVD indicating unfavorable prognosis of invasive ductal carcinoma (IDC) patients. Functionally, TGF-β1 promoted migration, proliferation and angiogenesis of differentiated-endothelial BCSLCs by inducing EndMT *in vitro* and promoted tumor growth and angiogenesis *in vivo*. Mechanically, we revealed TGF-β1 induced EndMT by activation of TGF-β and Notch signaling pathways with increase of p-Smad2/3 and Notch1 expression. Moreover, we found Snail and Slug were key factors of TGF-β and Notch signaling pathways.

**Conclusion:**

Our findings elucidated the mechanism of TGF-β1 in the promotion of angiogenesis and progression by EndMT in breast cancer.

## Introduction

Breast cancer is the leading cause of cancer-related mortality among women worldwide (1), accounting for more than 10% of the new cancer diagnoses each year (2). As early as 1971, Folkman (3) hypothesized that the growth of solid neoplasms is always accompanied by neovascularization.

Angiogenesis is a dynamic, continuous process of microvessel growth induced by tumor cells and the establishment of blood circulation in tumor. The classic tumor endothelial angiogenesis model proposed that tumor blood vessels are made up of normal endothelial cells. Moreover, our previous study found (4) that CCL18 enhanced the EndMT in normal human umbilical vein endothelial cells (HUVECs) and promoted endothelial cell migration and angiogenesis. In 1999, Maniotis et al. (5) presented a new tumor microcirculation model, vasculogenic mimicry (VM), which is the formation of vascular structures by the tumor endothelial cells themselves (6). Furthermore, Dennie et al. indicated that the mosaic blood vessels (7) that comprised both endothelial and tumor endothelial cells formed the luminal surface of the tumor blood vessels and were the intergradation between the VM and the classical endothelium-dependent vascular forms. Our previous study indicated (8) that endothelial progenitor cells were found in the initiating cells of breast tumors, which could differentiate into functional vascular endothelial cells. Therefore, understanding the specific process whereby the differentiation the endothelial cells disrupts the vascular basement membrane of the normal vascular is important.

EndMT is characterized by the loss of some cellular features of endothelial cells and gain of certain characteristics of mesenchymal cells (9). It is a multi-signal pathway with a multi-factorial process, including the TGF-β and Notch signaling pathways (10). Moreover, some vascular supporting cells may be endothelial cells that were transformed by the EndMT during the tube formation (11), which may indicate EndMT may promote tumor angiogenesis process and progression in cancer. Kokudo et al. (12) found that TGF-β1, which is a member of the growth factor superfamily (13), can promote the EndMT in embryonic stem cell-derived endothelial cells. In our study, we used the TGF-β1 to induce EndMT model of differentiated-endothelial BCSLCs and HUVECs.

In this study, we demonstrated that EndMT induced by TGF-β1 promoted angiogenesis and malignant behavior of breast cancer both *in vitro* and *in vivo.* The process was involved of activation in TGF-β and Notch signaling pathways, Snail and Slug were key factors.

## Materials and methods

### Patients and samples

Tumor tissues were collected from 86 IDC patients undergoing surgery in our hospital, archives between 2007 and 2016. Clinical parameters including age, sex, stage, pathological diagnosis, TNM status and recurrence were also collected. The tissues were fixed in the formalin and embedded in paraffin for pathological and immunohistochemical (IHC) analysis. All patients completed a telephone follow-up interview after the initial surgery, the follow up period ranged from 60 to 130 months. Our study protocol has been approved by the Ethics Committee of The First Affiliated Hospital, Shantou University Medical College. Informed consent was obtained from each enrolled patient.

### Immunohistochemistry (IHC) staining

The immunohistochemistry (IHC) and double-IHC staining were performed using a IHC kit (KIT9710, MXB) and a double stain IHC kit (ab210059, Abcam) according to the manual instructions. The staining intensity has been describe in the previous studies (14–19). The primary antibodies used in IHC were listed in **Table S6**.

### Cell culture

Human breast cancer cell MDA-MB-231 was obtained from the ATCC (American Type Culture Collection), HUVECs were isolated as described previously (20). All samples were collected with informed consent according to the Internal Review and the Ethics Boards of the First Affiliated Hospital, Shantou University Medical College.

### A TGF-β1-induced EndMT model of endothelial cell by treatment

We gathered the BCSLCs from the mammosphere-cultured MDA-MB-231 cells (21) and then induced an endothelial differentiation of the BCSLCs with VEGF (22), as described previously. The HUVECs and the differentiated-endothelial BCSLCs were cultured with endothelial cell basal medium. The experimental group was treated with TGF-β1 (Peprotech, USA) for 4–8 days.

### siRNA transfections and lentivirus transfection

BCSLCs and HUVECs were transfected with siRNA duplexes (Gene Pharma Shanghai) using Lipofectamine 2000 according to the manufacturer’s instructions. TGF-β1-shRNA-luc (5′-TTCTCCGAACGTGTCACGT-3′) (GenePharma Shanghai) was used to knockdown the expression of TGF-β1 in MDA-MB-231 according to the manufacturer’s instructions.

### Quantitative real-time PCR (qRT-PCR)

For qRT-PCR, total RNA extraction from all cell lines was performed using TRIzol reagent (TIANGEN, Beijing) and RNA was reversely transcribed to cDNA using PrimeScript™ RT Master Mix (Takara, Japan). qRT-PCR reactions were carried out using the qPCR SYBR Green Master Mix (Takara, Japan). The following primer sequences used for qRT-PCR were listed in **Table S7**.

The relative mRNA quantification was calculated using the 2−ΔΔCt method and normalized by GAPDH or β-actin. All the experiments were repeated in triplicate.

### Western blot analysis

Total cellular protein was separately extracted using RIPA buffer (Beyotime Biotechnology, China). In brief, the protein samples were separated using SDSPAGE gel (8–12%) electrophoresis and then transferred to PVDF membranes. After blockade in 5% skimmed milk, the membranes were incubated with primary antibodies at 4°C overnight followed by incubation of specific second antibodies for 1 h at room temperature. The target proteins were then detected using ECL reagent kits (Thermo, USA). The primary antibodies used in western blot were listed in **Table S8**.

### Wounding healing assay

The cells were plated in six-well plates and cultured until full confluence. Then suitable micropipette tips were used to generate uniformed scratches from the center of each well. After washed with phosphatebuffered saline (PBS), the cells were then cultured in fetal bovine serum (FBS)-free medium and taken photos at 0 h, 24h, 48 h and 72h. The cell migration was measured by comparing the gap change with the initial gap at 0 h. The experiment was performed independently in triplicate.

### Cell proliferation assays

Cell viability was measured with CCK-8 assay. For cell count assay, control and transfected cells were cultured in a 96-well plate (3000 cells/well). Triplicate wells were measured in each group. Cell viability was determined at 0h, 24h, 48h, 72h and 96h. The plate was incubated at 37°C for 2 h after each well was added with 10 μl CCK-8 solution (Dojindo, Japan). Then the spectrophotometric absorbance was measured at 450 nm for each sample.

### Matrigel tube-formation assay

The 96-well cell culture plate wells were coated evenly with Matrigel (BD, USA, 50μL). This was allowed to solidify at 37°C for 1 hour. The cells, treated with or without 10ng/mL TGF-β1, were then added (2.5 ×10^5^ cells/mL) onto the surface of the matrigel and incubated at 37°C for 12 hours. Vascular mimicry formation photographs were captured under the microscope, and the number of branch points were evaluated using Image J software.

### Nude mouse tumor-bearing model

Four-week-old female Balb/C-nu/nu nude mice, housed under standard conditions at the Center of Experimental Animal of Shantou University Medical College. Fifteen mice were randomly assigned to three groups and inoculated subcutaneously with MDA-MB-231 cells, NC-shRNA, and TGF-β1-ShRNA MDA-MB-231 cells (2 × 10^6^) in the fourth left mammary fat pad. Tumor growth rates were monitored weekly using digital calipers and a live animal imaging system with D-Luciferin potassium salt as the fluorescein. After eight weeks, the mice were sacrificed, and tumors were removed and collected for IHC and the dorsal skinfold window chamber (DSWC) model.

### Nude mouse DSWC model

BALB/C-nu/nu nude mice, with body weights ranging between 25–28g, were anaesthetized using 1% pentobarbital sodium and their skins were disinfected. Two identical window chambers were implanted on each side of the extended dorsal skinfold of the mice, forming a sandwiched double layer of the skin. Next, in a circular area of 12mm in diameter one layer of the skin together with the underlying muscle tissue was completely removed. The remaining layers, consisting of striated skin muscle (panniculus carnosus), subcutis, and cutis, were covered with a glass coverslip fixed with a snap ring on the frontside. After waiting for 72 hours for the mice to recover from the surgical trauma, the glass coverslip was removed. A “pocket” was created on the tissue with a needle. The tumor tissue from the nude mouse breast cancer model was then transplanted into the “pocket”, and covered with a glass coverslip and fixed with a snap ring. The branch points of the increased number of the blood vessels were measured using a dissecting microscope, and the growth rate of the tumors was monitored weekly using a live animal imaging system.

### Statistical analysis

SPSS software (version 21.0).was used for statistical analysis. Protein and RNA levels were compared using two-tailed Student’s *t* test. Correlations between MVD or EndMT markers expression in IDC tissues and clinicopathological features were analyzed with Pearson Chisquare (χ2) test. Desease free survival (DFS) and overall survival (OS) was assessed by Kaplan-Meier method, difference between survival curves was determined by logrank test. Differences with a *p* value < 0.05 were considered as statistically significant.

## Results

### High expression of MVD predicted unfavorable prognosis of IDC patients

**Figure 1:**
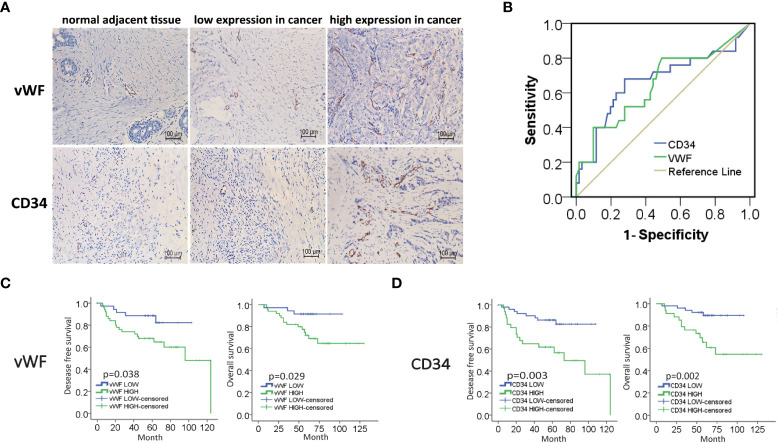
High expression of MVD predicted unfavorable prognosis of IDC patients. **(A)** Representative images of immunohistochemical (IHC) staining of vWF and CD34 in samples from breast invasive ductal carcinoma (IDC) patients. **(B)** ROC curve of the CD34 and vWF by the DFS of IDC patients. **(C, D)** Kaplan-Meier analysis of DFS and OS of IDC patients from our cohort by vWF and CD34.

To investigate the potential role of neovascularization in breast cancer, we first performed IHC staining for MVD (CD34, vWF), an important quantitation of the neovascularization, on samples from 86 IDC patients (**Figure 1A**). The ROC curve of desease free survival (DFS) was used to determine the cutoff point of MVD (**Figure 1B** and **Table S1**). The correlation analysis of MVD levels with clinicopathological features of enrolled IDC patients were performed. As shown, high expression of MVD was positively correlated with lymphatic invasion, remote metastasis and negative hormone receptor (**Table 1**), indicating MVD might play an important role in modulating malignant behavior of breast cancer. Moreover, Kaplan-Meier analysis revealed that patients with high MVD expression exhibited unfavorable DFS (**Figure 1C**) and overall survival (OS, **Figure 1D**). In addition, multivariate analysis showed that MVD was an independent risk factor for DFS and OS of IDC patients from our cohort (**Tables S2**, **S3**). These results suggested MVD was a reliable prognostic marker of IDC patients.

**Table 1:** Relationship between the expression of CD34, vWF and clinicopathologic parameters of IDC patients.

Variables	Case (N=86)	CD34 level		P value	vWF level		P value
		LOW (n=52)	HIGH (n=34)		LOW (n=36)	HIGH (n=50)	
Age (years)				0.429			0.166
<50	41	23 (44.2)	18 (52.9)		14 (38.9)	27 (54.0)	
≥	45	29 (55.8)	16 (47.1)		22 (61.1)	23 (46.0)	
TNM stage				0.090			0.614
I	21	16 (30.8)	5 (14.7)		10 (27.8)	11 (22.0)	
II-III	65	36 (69.2)	29 (85.3)		26 (72.2)	39 (78.0)	
T classification				0.123			0.468
T1	23	17 (32.7)	6 (17.6)		8 (22.2)	15 (30.0)	
T2-T4	63	35 (67.3)	28 (82.4)		28 (77.8)	35 (70.0)	
Lymphatic invasion				0.020			0.039
N0	51	36 (69.2)	15 (44.1)		26 (72.2)	25 (50.0)	
N1-3	35	16 (30.8)	19 (55.9)		10 (27.8)	25 (50.0)	
Histological grading				0.719			0.744
Well+ moderate	46	27 (51.9)	19 (55.9)		20 (55.6)	26 (52.0)	
Poor	40	25 (48.1)	15 (44.1)		16 (44.4)	24 (48.0)	
Hormone receptor				0.156			0.011
Negative	45	24 (46.2)	21 (61.8)		13 (36.1)	32 (64.0)	
Positive	41	28 (53.8)	13 (38.2)		23 (63.9)	18 (36.0)	
Metastatic and relapse sites							
Lung		5 (9.6)	11 (32.4)	0.008	3 (8.3)	13 (26.0)	0.038
Bone		2 (3.8)	7 (20.6)	0.013	1 (2.8)	8 (16.0)	0.048
Liver		3 (5.8)	6 (17.6)	0.079	2 (5.6)	7 (14.0)	0.207
Chest wall		1 (1.9)	4 (11.8)	0.057	1 (2.8)	4 (8.0)	0.307
Brain		0 (0.0)	3 (8.8)	0.029	0 (0.0)	3 (6.0)	0.135

### MVD level is positively correlated with the level of EndMT markers in breast cancer

**Figure 2:**
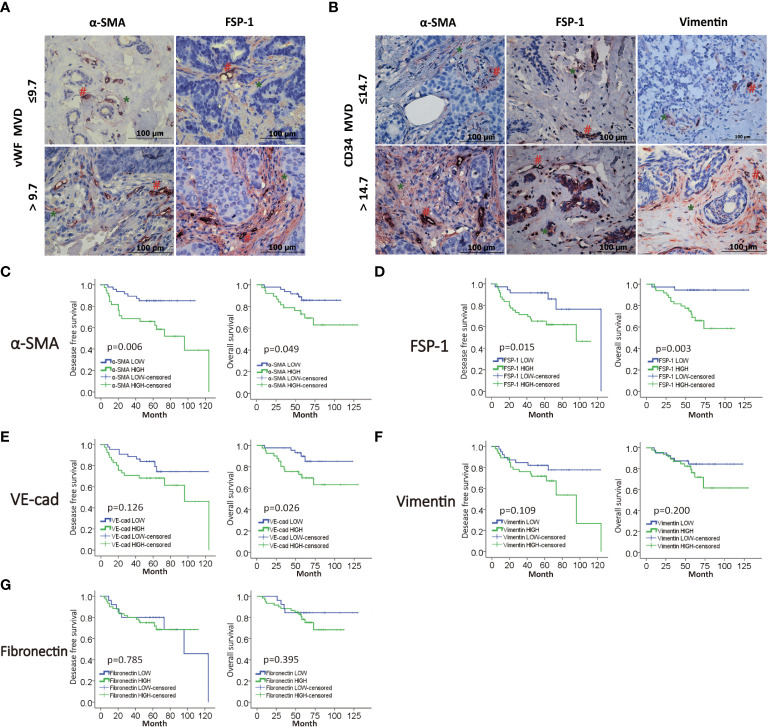
MVD level is positively correlated with EndMT markers in breast cancer. **(A,B)** Representative images of double-IHC staining of vWF, CD34 (brown, with red “#”) and either α-SMA, FSP-1 or Vimentin (red, with green “*”) in samples from IDC patients. **(C–G)** Kaplan-Meier analysis of DFS and OS of IDC patients from our cohort by α-SMA, FSP-1, Vimentin, VE-Cadherin and Fibronectin.

We next planned to study the role of EndMT in angiogenesis of breast cancer, then we performed double-IHC staining for EndMT markers (α-SMA, FSP-1, Vimentin, VE-Cadherin and Fibronectin), and MVD (CD34, vWF) on IDC samples. Chi-square test was performed to identify the correlation between vWF and CD34, and the *P* value of less than 0.000, which meant that most of the low expression cases and high expression cases between CD34 and vWF overlap (**Table S4**). As shown, the expression of α-SMA, FSP-1 and Vimentin was positively correlated with MVD level (**Figures 2A, B**, and **Table S5**). Kaplan-Meier analysis revealed that patients with high level of EndMT exhibited unfavorable DFS and OS (**Figures 2C-G**). With above evidence, we speculated that EndMT may promote tumor angiogenesis and cause poor clinical outcome of breast cancer.

### TGF-β1 promoted migration, proliferation and angiogenesis of BCSLCs by inducing EndMT

**Figure 3:**
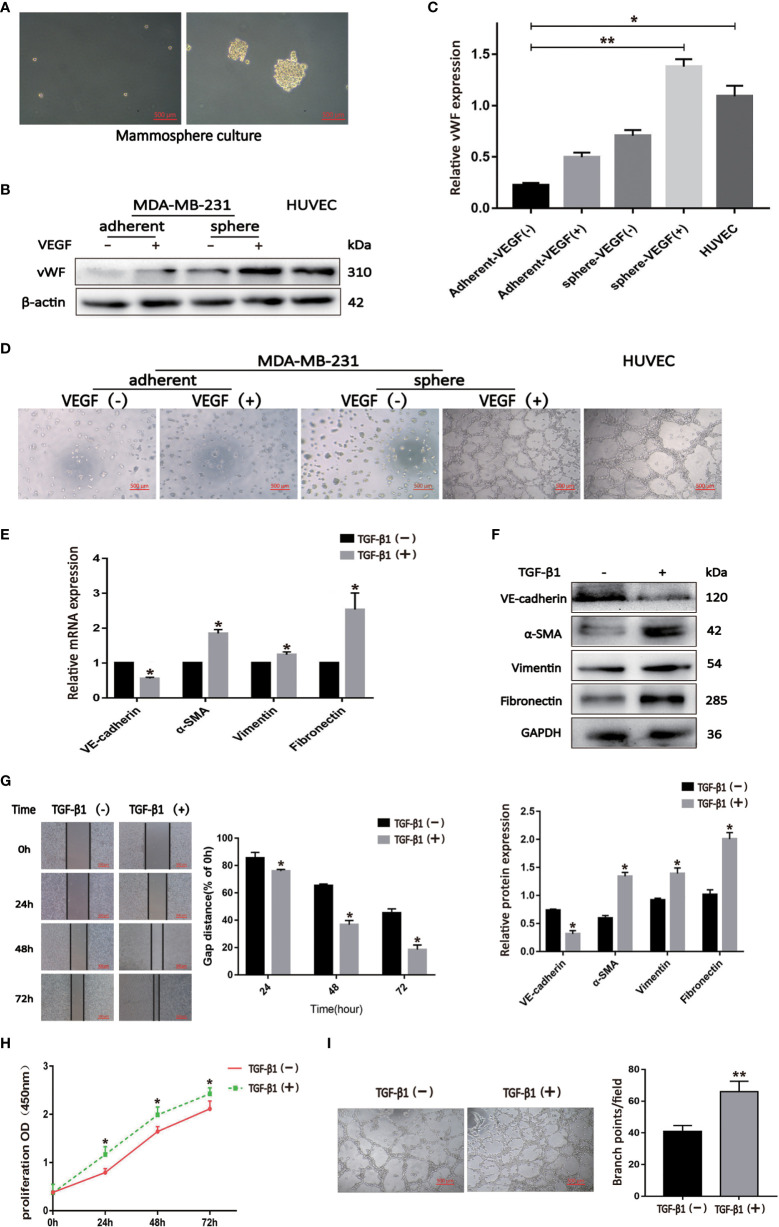
TGF-β1 promoted migration, proliferation and angiogenesis of BCSLCs by inducing EndMT. **(A)** Image of BCSLCs. **(B)** Western blot was performed to measure the vWF expression. **(C)** Protein band intensity of vWF was quantified by western blot. *p<0.05 and **p<0.01 versus Adherent-VEGF(-) group. **(D)** Representative images of capillary-like structures on matrigel formed by adherent or mammosphere-cultured MDA-MB-231 treated with or without VEGF. **(E, F)** The mRNA and protein expression of EndMT markers in differentiated-endothelial BCSLCs treated with or without TGF-β1. **(G)** Cell scratch wound-healing assay was performed to measure the migration of differentiated-endothelial BCSLCs treated with or without TGF-β1. **(H)** CCK-8 assay was performed to measure the proliferation of differentiated-endothelial BCSLCs treated with or without TGF-β1. **(I)** Matrigel tube-formation assay was performed to measure the tube-formation ability of differentiated-endothelial BCSLCs treated with or without TGF-β1. *p < 0.05 and **p< 0.01 versus TGF-β(-) group. All data were representative of three independent experiments. Means ± SD were shown.

We cultured BCSLCs using a mammosphere culture with 10 ng/ml VEGF treatment, and VEGF-induced BCSLCs had increased expression of vWF and developed capillary-like structures in the matrigel as HUVECs, suggesting that BCSLCs were successfully induced into differentiated-endothelial BCSLCs (**Figures 3A–D**). To define the functional roles of EndMT in breast cancer, TGF-β1 was used to induce EndMT in differentiated-endothelial BCSLCs. The expression of VE-Cadherin was decreased, while the expression of α-SMA, Vimentin and Fibronectin was increased by qRT-PCR and western blot, indicating EndMT in differentiated-endothelial BCSLCs was induced (**Figures 3E, F**). The promotion of migration, proliferation and angiogenesis of differentiated-endothelial BCSLCs cells was then evaluated by wound healing assay, cell proliferation assay and matrigel tube-formation assay, respectively (**Figures 3G–I**). Similarly, we treated HUVECs with TGF-β1, and we found EndMT was enhanced in HUVECs with promotion of migration, proliferation and tube-formation ability (**Figures S1A-F**). These results suggested TGF-β1 promoted migration, proliferation and angiogenesis of BCSLCs by inducing EndMT.

### EndMT is regulated by TGF-β and Notch signaling pathways in BCSLCs

**Figure 4:**
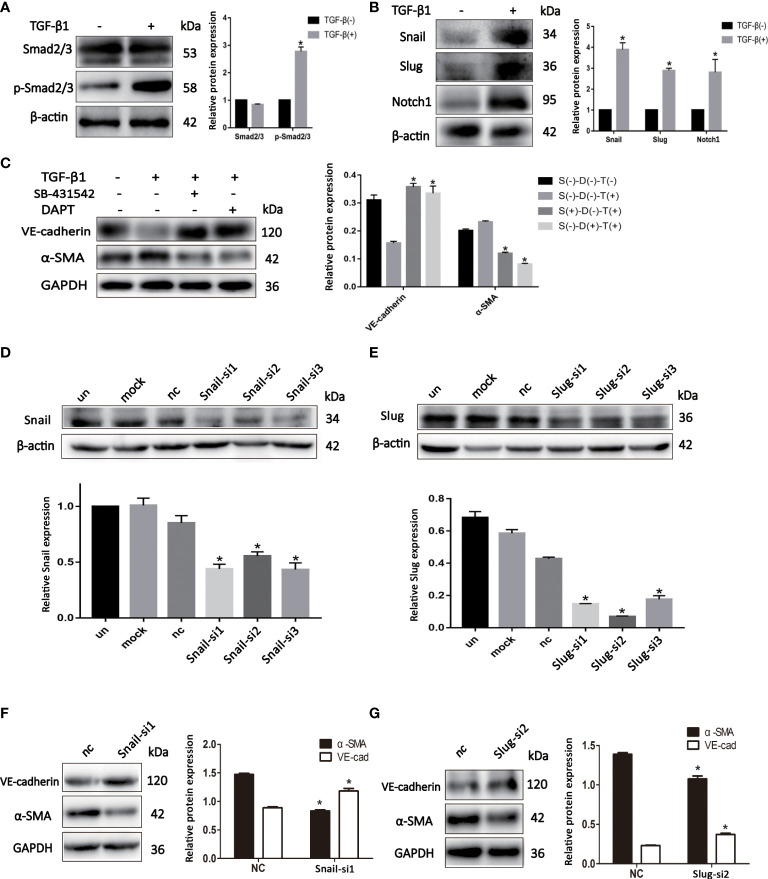
EndMT is regulated by TGF-β and Notch signaling pathways in BCSLCs. **(A, B)** The protein expression of TGF-β and Notch signaling pathways markers in differentiated-endothelial BCSLCs treated with or without TGF-β1. **(C)** The protein expression of EndMT markers in differentiated-endothelial BCSLCs treated with SB-431542 or DAPT. *p < 0.05 versus S(-)-D(-)-T(-) group. **(D, E)** Knockdown of Snail and Slug were confirmed by western blot in differentiated-endothelial BCSLCs. *p < 0.05 versus un group. **(F, G)** The protein expression of EndMT markers in differentiated-endothelial BCSLCs with si-Snail and si-Slug. *p < 0.05 versus NC group. All data were representative of three independent experiments. Means ± SD were shown.

We sought to investigate the mechanism by which EndMT promoted malignant phenotype of BCSLCs. The expression of p-Smad2/3 and Notch1 was increased in BCSLCs treated with TGF-β1 (**Figures 4A, B**). Inhibitors of TGF-β (SB-431542, 10μM) and Notch (DAPT, 16μM) signaling pathway were used to determine whether they could inhibit EndMT in BCSLCs. As shown, the expression of VE-Cadherin was increased, while the expression of α-SMA was decreased (**Figure 4C**). In addition, the expression of p-Smad2/3 and Notch1 was also increased in HUVECs treated with TGF-β1 (**Figures S2A, B**). Besides, we used two inhibitors above to treat HUVECs, the inhibition of EndMT in HUVECs was also observed (**Figure S2C**) with attenuation of migration, proliferation and angiogenesis (**Figures S2D-I**). These results suggested EndMT was regulated by TGF-β and Notch signaling pathways.

Subsequently, we knockdowned the expression of Snail and Slug in BCSLCs with Snail siRNA and Slug siRNA (**Figures 4D, E**), EndMT was inhibited (**Figures 4F, G**). Likewise, inhibition of HUVECs with Snail siRNA and Slug siRNA was found (**Figures S2J–M**). Furthermore, migration, proliferation and angiogenesis of HUVECs was inhibited when Snail and Slug were knockdown (**Figures S2N–S**). Collectively, these results suggested EndMT was regulated by TGF-β and Notch signaling pathways in BCSLCs, Snail and Slug were key regulatory factors in both TGF-β and Notch signaling pathways.

### TGF-β1 promoted tumor growth and angiogenesis of breast cancer *in vivo*

**Figure 5:**
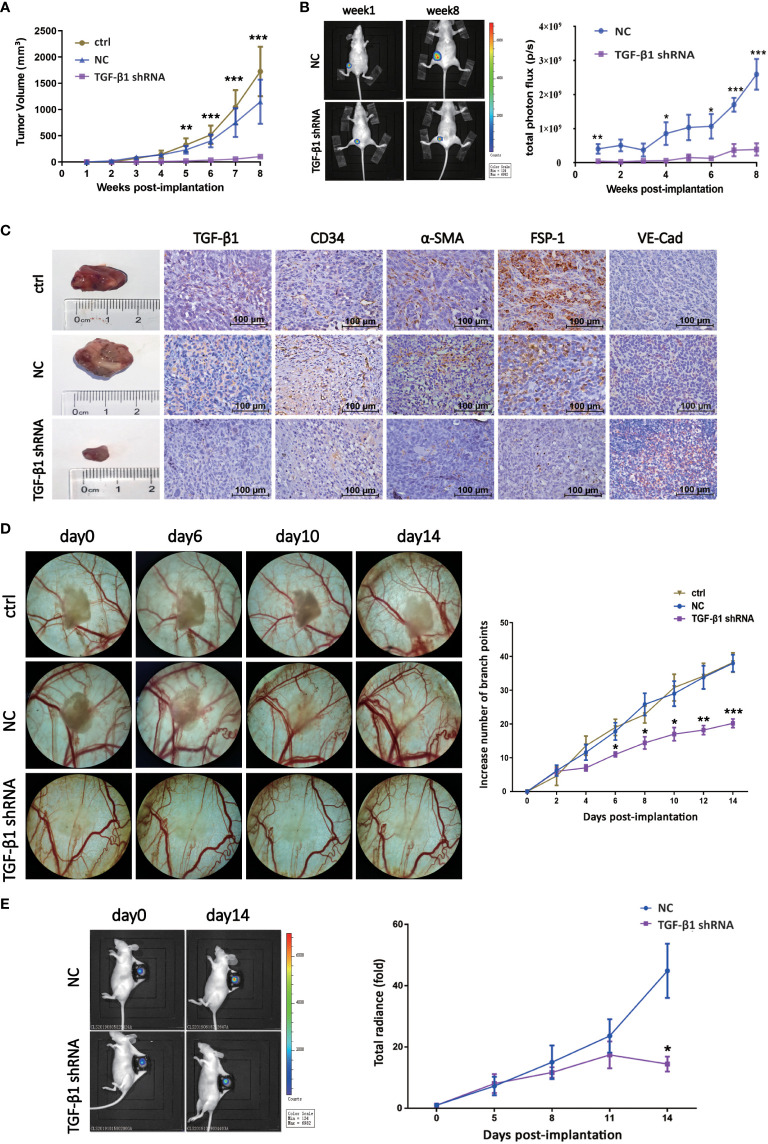
TGF-β1 promoted tumor growth and angiogenesis of breast cancer *in vivo*. **(A)** Quantitative analysis of tumor volume of xenografts. Tumor volume was compared at indicated time points. **p < 0.01 and ***p < 0.001 versus ctrl group. **(B)** Representative pictures of luciferase imaging and quantitative analysis of total photon flux of xenografts. Total photon flux was compared at indicated time points. *p < 0.05, **p < 0.01 and *p < 0.001 versus NC group. **(C)** Representative images of xenografts and IHC staining for TGF-β1, CD34, α-SMA, FSP-1 and VE-Cadherin were shown. **(D)** Representative images of blood vessels of DSWC model and quantitative analysis of branch points. Branch point was compared at indicated time points. *p < 0.05, **p < 0.01 and *p < 0.001 versus ctrl group. **(E)** Representative pictures of luciferase imaging and quantitative analysis of total photon flux of xenografts. Total photon flux was compared at indicated time points. *p < 0.05 versus NC group. Means ± SD were shown.

In order to explore the effect of TGF-β1 on tumor growth and angiogenesis of breast cancer *in vivo*, we first established nude mouse tumor-bearing model with TGF-β1 knockdown breast cancer cells (**Figures S3A, B**. We collected the data of tumor volume and images at different time point to dynamically observe enlargement of tumor. As shown, knockdown of TGF-β1 inhibited tumor growth of breast cancer (**Figures 5A-C**). Besides, the expression of VE-Cadherin was increased, while the expression of α-SMA was decreased, which meant knockdown of TGF-β1 inhibited EndMT in breast cancer (**Figure 5C**).

Then, nude mouse DSWC model was constructed to observe the effect of TGF-β1 on angiogenesis of breast cancer *in vivo*, and schematic diagram of skin anatomy and timeline of animal experiment were shown (**Figures S3C, D**). Loss of TGF-β1 resulted in decrease of branch points and tumor radiance (**Figures 5D, E**), indicating angiogenesis and tumor growth of breast cancer were inhibited. These results suggested TGF-β1 promoted tumor growth and angiogenesis of breast cancer *in vivo*.

## Discussion

We analyzed MVD through CD34 and vWF, and we found that CD34 and vWF had obvious correlation in breast cancer tissues, which meant that these two indicators could reflect MVD in breast cancer. In our immunohistochemical and clinical data analysis, higher MVD expression levels were associated with poor clinicopathological characteristics such as greater lymphatic invasion and remote metastasis. The results of this study suggested that tumor angiogenesis may be an important factor in the promotion of malignant behavior of tumors. Furthermore, we found that the expression of mesenchymal markers such as α-SMA, vimentin, and FSP1 were proportional to the level of MVD in the samples from IDC patients. Magrini et al. (23) found that the neural adhesion molecule L1 can promote EndMT, increase angiogenesis, and decrease vascular stabilization, leading to tumor growth and metastasis in the orthotopic mouse model of pancreatic carcinoma, which was consistent with our results.

To confirm that EndMT is associated with tumor angiogenesis in IDC patients, we further investigated possible molecular mechanisms. Tumor blood vessels can be made up of normal endothelial and differentiated cancer stem cells. In this study, TGF-β1 was used to stimulate differentiated-endothelial BCSLCs and normal endothelial cell, then the cells transformed their morphology from endothelial to mesenchymal cells. Our results were consistent with those of other reports (24, 25). The EndMT group showed greater cell proliferation, migration, and angiogenesis ability than the control group in both normal endothelial cells and BCSLCs; in addition studies of systemic sclerosis (26) and silicosis (27) had found similar results. The data indicated that TGF-β1 promoted EndMT in differentiated-endothelial BCSLCs, which resulted in new blood vessel formation, showing that an important portion of the tumor vascular endothelium came from cancer cells and not from normal endotheliocytes.

Accumulating evidence (28, 29) has highlighted the critical role of the TGF-β or Notch signaling pathway in EndMT. Notch1 can regulate EndMT in cardiac cushions and TGF-β1 can promote endothelial fibrosis under inflammatory conditions *via* the promotion of EndMT. Moreover, Notch1 and TGF-β1 can upregulate the expression of Snail and Slug during EndMT (30, 31). Our data demonstrated that the inhibitors of the TGF-β or Notch signaling pathway can inhibit EndMT in differentiated-endothelial BCSLCs and HUVECs. We also knockdown the expression of Slug and Snail in differentiated-endothelial BCSLCs and HUVECs, and we found that the loss of Snail and Slug in these two cells could efficiently inhibit EndMT with TGF-β1 treatment. These findings suggested that TGF-β1 regulated EndMT in breast cancer *via* the TGF-β and Notch signaling pathways, and Snail and Slug may be key factors, implying that strategies which simultaneously inhibit TGF-β1, Snail and Slug may improve the effects of anti-angiogenic therapies in breast cancer.

The dorsal skinfold window chamber model (DSWC) is one of the most effective methods for observing the growth of the blood vessels *in vivo*. Compared with the commonly used *in vivo* angiogenesis experiments, such as chicken chorioallantoic membrane model (CAM model) and corneal neovascularization (CNV model), the DSWC model allows real-time dynamic monitoring of blood vessels, observation of the vascular microcirculation, and analysis the various aspects of tumor biology and treatment responses.

Animal experiments showed that tumor progression and angiogenesis of breast cancer was inhibited when the TGF-β1 level and EndMT process of the tumor was downregulated. This is consistent with the results of previous studies that TGF-β1 can induce angiogenesis in mouse colon tumors (32). In conclusion, our data described a novel function of EndMT in angiogenesis in breast cancer both *in vitro* and *in vivo*. Thus, EndMT and its potential key factors TGF-β1, Snail and Slug may serve as therapeutic targets for inhibiting angiogenesis in breast cancer.

To our knowledge, this is the first report showing that TGF-β1-induced EndMT *via* TGF-β and notch signaling pathways can promote tumor angiogenesis in both differentiated-endothelial BCSLCs and normal endothelial cells in breast cancer. Our findings suggest that the inhibition of EndMT progress may sensitize tumors to anticancer therapies and help overcome resistance to anti-angiogenic therapies.

## Data availability statement

The original contributions presented in the study are included in the article/**Supplementary Material**. Further inquiries can be directed to the corresponding authors.

## Ethics statement

The studies involving human participants were reviewed and approved by the ethics committee of The First Affiliated Hospital, Shantou University Medical College (No. [2016]-026). The animal study was reviewed and approved by the Animal ethics committee of Shantou University Medical College (No. SUMC [2016]-081).

## Author contributions

LL and Y-SC conceived and designed the experiments. Z-XL and X-BY performed the IHC experiments. Z-JZ, W-JC, and YG performed the cell experiments. J-XC, Z-XL, and Y-YH performed the animal experiments. J-X C, Z-X L, FX, Y-S C, and LL analyzed the data. J-XC, Z-XL, Y-SC, and LL wrote the paper. All authors reviewed the manuscript. All authors read and approved the final manuscript.

## Funding

This study was supported by grants from National Natural Science Foundation of China (No. 81672640 to LL), Basic and Applied Basic Research Foundation of Guangdong Province (No. 2021A1515010137 to LL), the Special Funds for science and technology of Guangdong Province (No. 2021-88 to LL and Y-SC) and the Project of Innovating and Strengthening Universities in Guangdong Province (No. 2020KQNCX021 to Z-XL).

## Acknowledgments

We thank the Laboratory of Molecular Cardiology, First Affiliated Hospital, Shantou University Medical College, for supplying the laboratory. We also thank L-B W, B-Z C and XZ for their technical assistance.

## Conflict of interest

The authors declare that the research was conducted in the absence of any commercial or financial relationships that could be construed as a potential conflict of interest.

## Publisher’s note

All claims expressed in this article are solely those of the authors and do not necessarily represent those of their affiliated organizations, or those of the publisher, the editors and the reviewers. Any product that may be evaluated in this article, or claim that may be made by its manufacturer, is not guaranteed or endorsed by the publisher.

## Supplementary material

Supplementary Figure 1
**(A-B)** The protein expression of EndMT markers in HUVECs treated with or without TGF-β1. **(C-F)** Cell scratch wound-healing assay, transwell migration assay, cell count assay, and matrigel tube-formation assay were performed to measure the migration, proliferation and tube-formation ability of HUVECs treated with or without TGF-β1, respectively. *p < 0.05, **p < 0.01 and *p < 0.001 versus ctrl group.

Supplementary Figure 2
**(A-B)** The protein expression of TGF-β and Notch signaling pathways markers in HUVECs treated with or without TGF-β1. *p < 0.05 versus TGF-β(-) group. **(C)** The protein expression of EndMT markers in HUVECs treated with SB-431542 or DAPT. **(D-I)** Transwell migration assay, cell count assay, and matrigel tube-formation assay were performed to measure the migration, proliferation and tube-formation ability of HUVECs treated with or without SB-431542 and DAPT. *p < 0.05, **p < 0.01 and *p < 0.001 versus DMSO group. **(J-K)** Knockdown of Snail and Slug were confirmed by western blot in HUVECs. *p < 0.05, **p < 0.01 and *p < 0.001 versus un group. **(L-M)** The protein expression of EndMT markers in HUVECs with si-Snail and si-Slug. **(N-S)** Transwell migration assay, cell count assay, and matrigel tube-formation assay were performed to measure the migration, proliferation and tube-formation ability of HUVECs with si-Snail and si-Slug. *p < 0.05, **p < 0.01 and *p < 0.001 versus NC group.

Supplementary Figure 3
**(A)** Knockdown of TGF-β1 was confirmed by western blot in MDA-MB-231. ***p < 0.001 versus ctrl group. **(B)** Process of nude mouse breast cancer tumor-bearing model. **(C)** Process of nude mouse DSWC model. **(D)** Schematic diagram of skin anatomy.
