# Corrigendum: TGF-β1 promotes human breast cancer angiogenesis and malignant behavior by regulating endothelial-mesenchymal transition

**DOI:** 10.3389/fonc.2022.1118572

**Published:** 2023-01-10

**Authors:** Zi-Xiong Li, Jie-Xin Chen, Ze-Jun Zheng, Wang-Jing Cai, Xiong-Bin Yang, Yuan-Yuan Huang, Yao Gong, Feng Xu, Yong-Song Chen, Ling Lin

**Affiliations:** ^1^ Department of Rheumatology and Immunology, The First Affiliated Hospital of Shantou University Medical College, Shantou, China; ^2^ Department of Thyroid and Breast Surgery, The First Affiliated Hospital of Shantou University Medical College, Shantou, China; ^3^ Department of Rheumatology, Shantou University Medical College, Shantou, China; ^4^ Department of Respiratory and Critical Care Medicine, The First Affiliated Hospital of Shantou University Medical College, Shantou, China; ^5^ Department of Endocrinology, The First Affiliated Hospital of Shantou University Medical College, Shantou, China

**Keywords:** TGF-β1, EndMT, angiogenesis, breast cancer, BCSLC, dorsal skinfold window chamber

**Error in Figure/Table**

In the published article, there was an error in **Figure 1** High expression of MVD predicted unfavorable prognosis of IDC patients. as published. In **Figure 1B**, the title of X axis was “Specificity”, it should be corrected to “1-Specificity”. The corrected **Figure 1** High expression of MVD predicted unfavorable prognosis of IDC patients. and its caption appear below.

**Figure 1:**
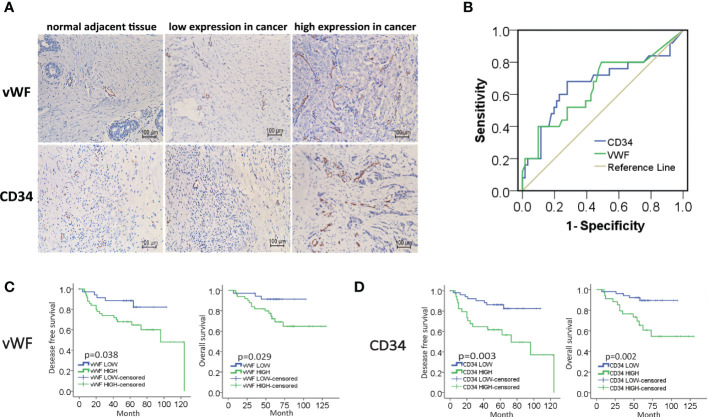
High expression of MVD predicted unfavorable prognosis of IDC patients. **(A)** Representative images of immunohistochemical (IHC) staining of vWF and CD34 in samples from breast invasive ductal carcinoma (IDC) patients. **(B)** ROC curve of the CD34 and vWF by the DFS of IDC patients. **(C, D)** Kaplan-Meier analysis of DFS and OS of IDC patients from our cohort by vWF and CD34.

In the published article, there was an error in **Figure 2** MVD level is positively correlated with EndMT markers in breast cancer. as published. In **Figure 2A, B**, the red “#” and green “*” were missing. The corrected **Figure 2** MVD level is positively correlated with EndMT markers in breast cancer. and its caption appear below.

**Figure 2:**
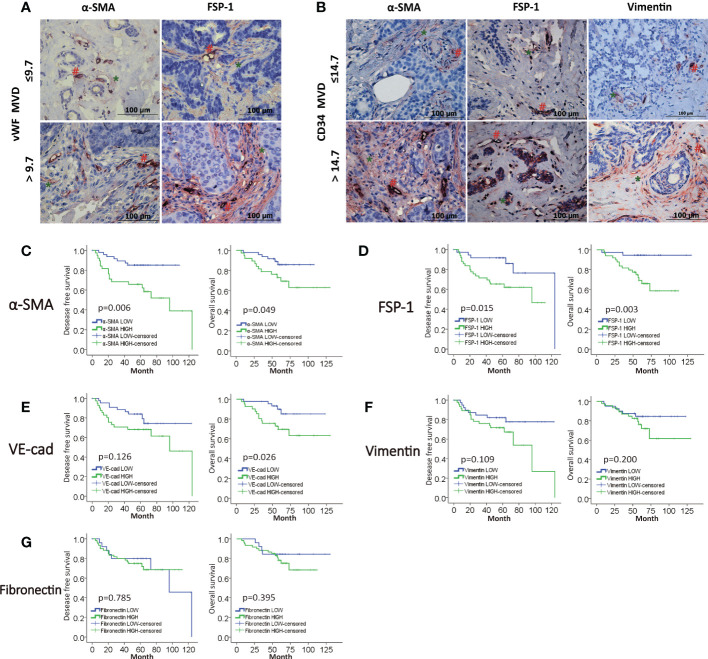
MVD level is positively correlated with EndMT markers in breast cancer. **(A, B)** Representative images of double-IHC staining of vWF, CD34 (brown, with red “#”) and either α-SMA, FSP-1 or Vimentin (red, with green “*”) in samples from IDC patients. **(C–G)** Kaplan-Meier analysis of DFS and OS of IDC patients from our cohort by α-SMA, FSP-1, Vimentin, VE-Cadherin and Fibronectin.

The authors apologize for these errors and state that they do not change the scientific conclusions of the article in any way. The original article has been updated.

