# Supplementary figures and images for: TGF-β1 promotes human breast cancer angiogenesis and malignant behavior by regulating endothelial-mesenchymal transition
Source: Front Oncol. 2022 Nov 16;12:1051148. doi: 10.3389/fonc.2022.1051148 (PMC9709251; doi:10.3389/fonc.2022.1051148)

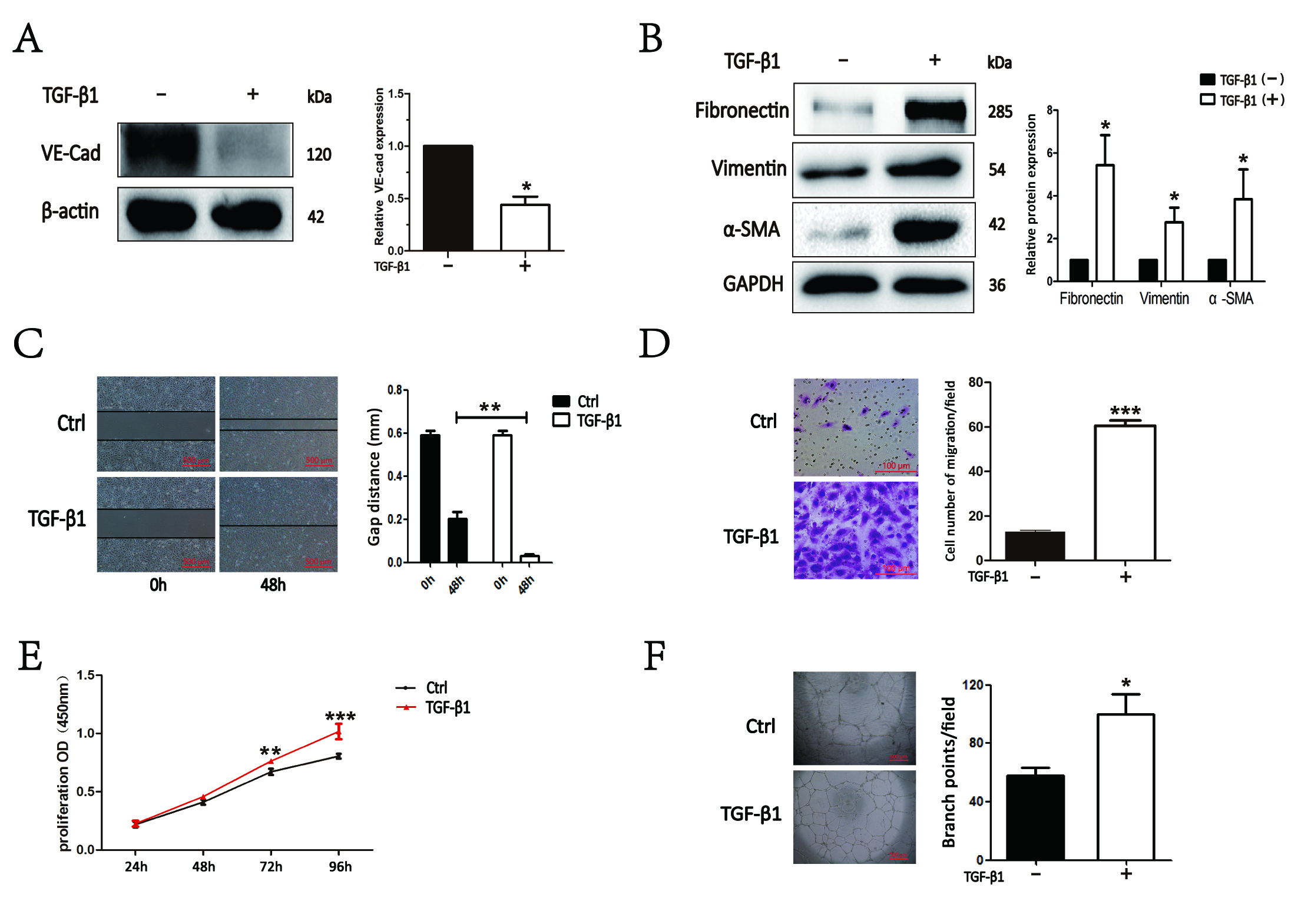

Supplement: Supplementary Figure 1 — (A-B) The protein expression of EndMT markers in HUVECs treated with or without TGF-β1. (C-F) Cell scratch wound-healing assay, transwell migration assay, cell count assay, and matrigel tube-formation assay were performed to measure the migration, proliferation and tube-formation ability of HUVECs treated with or without TGF-β1, respectively. *p < 0.05, **p < 0.01 and *p < 0.001 versus ctrl group. [file Image_1.jpg]

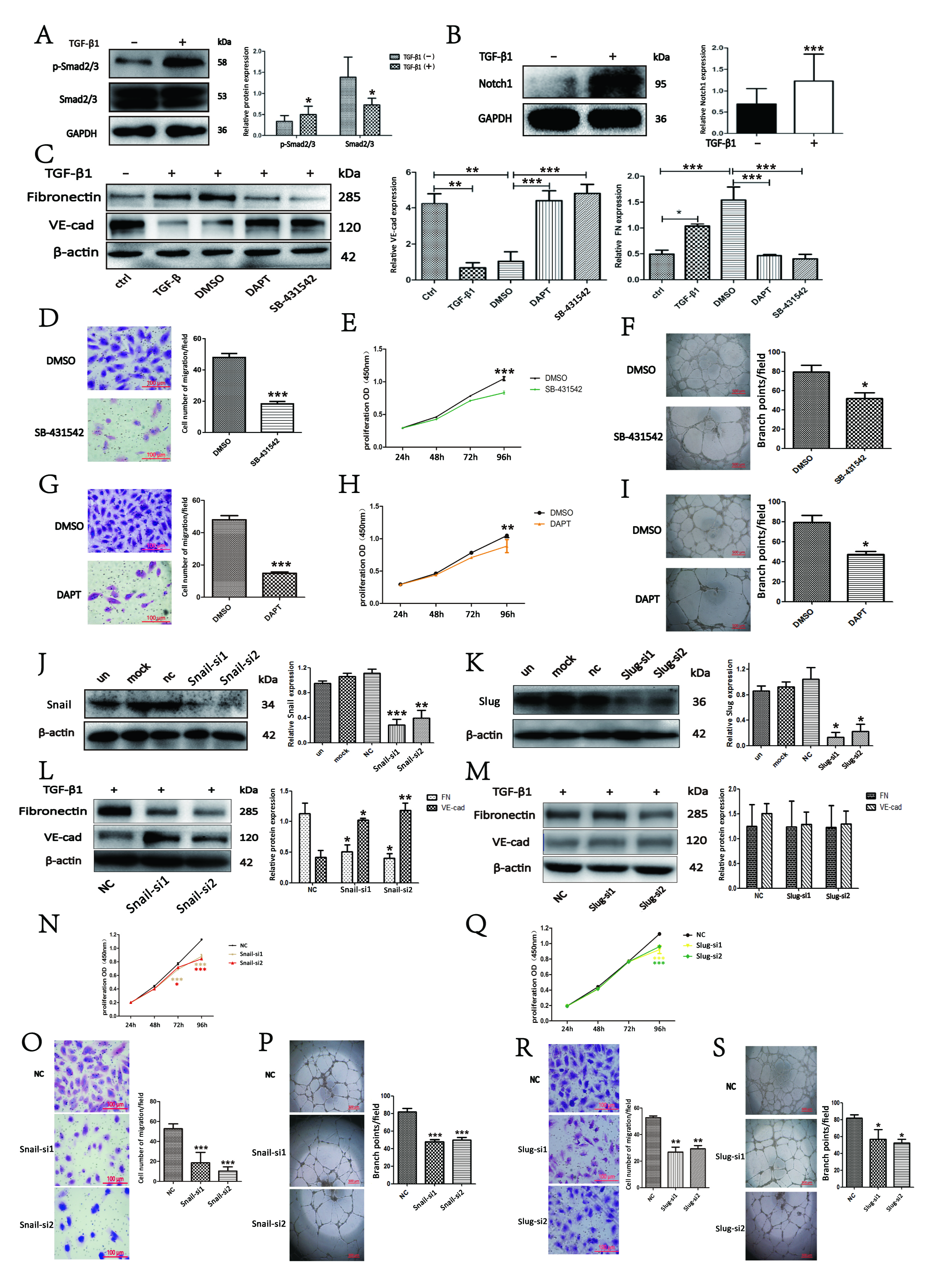

Supplement: Supplementary Figure 2 — (A-B) The protein expression of TGF-β and Notch signaling pathways markers in HUVECs treated with or without TGF-β1. *p < 0.05 versus TGF-β(-) group. (C) The protein expression of EndMT markers in HUVECs treated with SB-431542 or DAPT. (D-I) Transwell migration assay, cell count assay, and matrigel tube-formation assay were performed to measure the migration, proliferation and tube-formation ability of HUVECs treated with or without SB-431542 and DAPT. *p < 0.05, **p < 0.01 and *p < 0.001 versus DMSO group. (J-K) Knockdown of Snail and Slug were confirmed by western blot in HUVECs. *p < 0.05, **p < 0.01 and *p < 0.001 versus un group. (L-M) The protein expression of EndMT markers in HUVECs with si-Snail and si-Slug. (N-S) Transwell migration assay, cell count assay, and matrigel tube-formation assay were performed to measure the migration, proliferation and tube-formation ability of HUVECs with si-Snail and si-Slug. *p < 0.05, **p < 0.01 and *p < 0.001 versus NC group. [file Image_2.jpg]

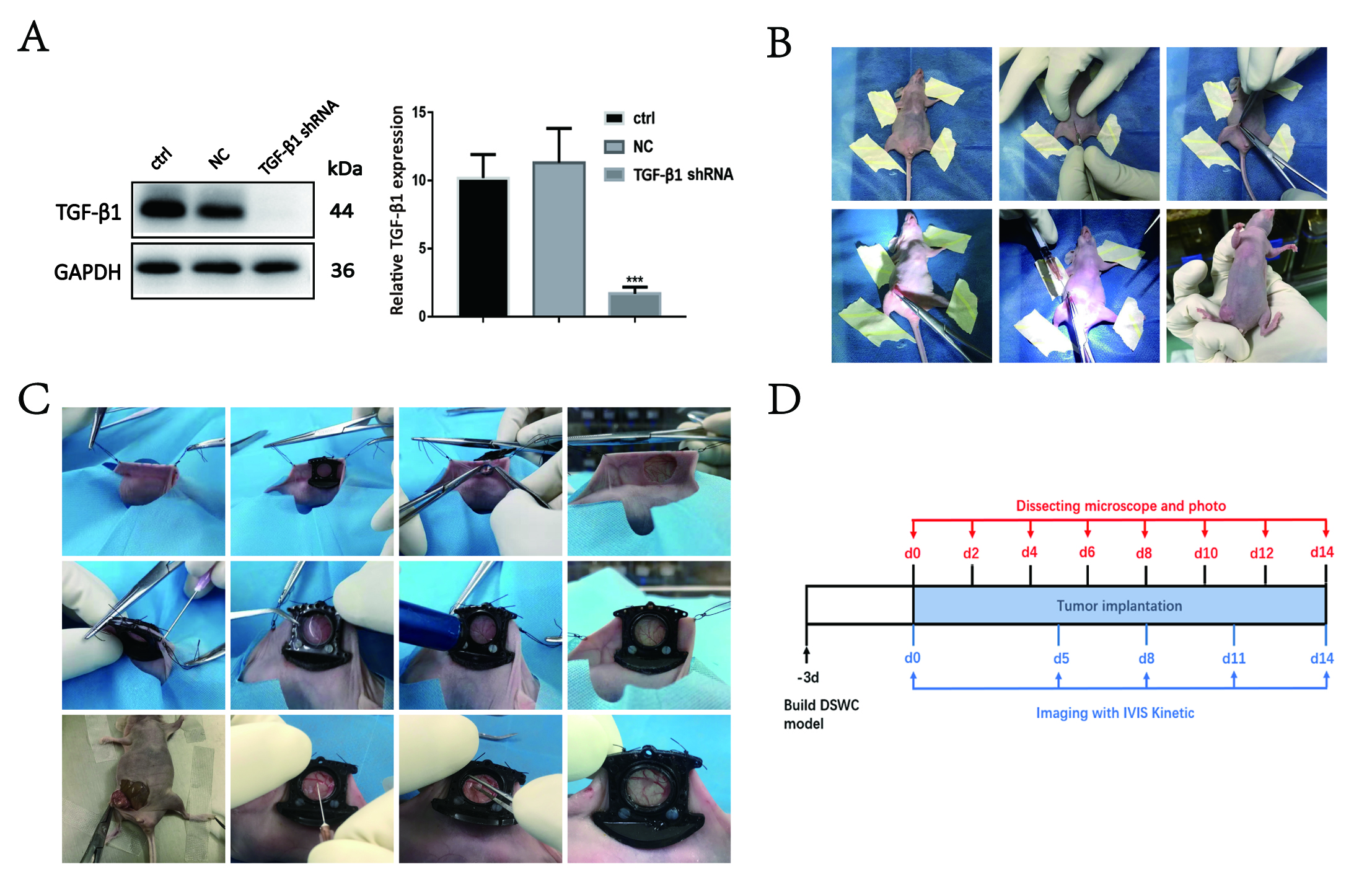

Supplement: Supplementary Figure 3 — (A) Knockdown of TGF-β1 was confirmed by western blot in MDA-MB-231. ***p < 0.001 versus ctrl group. (B) Process of nude mouse breast cancer tumor-bearing model. (C) Process of nude mouse DSWC model. (D) Schematic diagram of skin anatomy. [file Image_3.jpg]
