# Supplementary material for: TGF-β1 promotes human breast cancer angiogenesis and malignant behavior by regulating endothelial-mesenchymal transition
Source: Front Oncol. 2022 Nov 16;12:1051148. doi: 10.3389/fonc.2022.1051148 (PMC9709251; doi:10.3389/fonc.2022.1051148)
Supplement: Supplementary file 4 [file DataSheet_1.zip › Supplementary Table 1.DOC]

**Table S1** Relationship between CD34, vWF and DFS analyzed by ROC

|  | DFS | | |
| --- | --- | --- | --- |
|  | Area | CI(95%) | Cut-off point |
| CD34 | 0.666 | 0.525-0.806 | 14.3 |
| vWF | 0.648 | 0.511-0.784 | 9.7 |
