# Supplementary material for: TGF-β1 promotes human breast cancer angiogenesis and malignant behavior by regulating endothelial-mesenchymal transition
Source: Front Oncol. 2022 Nov 16;12:1051148. doi: 10.3389/fonc.2022.1051148 (PMC9709251; doi:10.3389/fonc.2022.1051148)
Supplement: Supplementary file 4 [file DataSheet_1.zip › Supplementary Table 2.DOC]

**Table S2** Univariate and multivariate analysis of clinicopathologic factors for DFS of IDC patients in our cohort

| Factor | DFS | | | |  |
| --- | --- | --- | --- | --- | --- |
|  | Univariate | | Multivariate | |  |
|  | HR (95%CI) | P value | HR (95%CI) | P value |  |
| Age (＜50 vs ≥50)  Tumor size  (＜2cm vs≥2cm)  Lymphatic invasion  (Yes vs no )  TNM (I vs II-III)  CD34 (Low vs High)  vWF (Low vs High) | 1.711(0.732-4.000)  4.909(1.152-20.93)  4.323(1.780-10.50)  8.638(1.166-64.01)  3.410(1.451-8.015)  2.734(1.014-7.371) | 0.215  0.032*  0.005*  0.035*  0.005*  0.047* | /  /  3.431(1.380-8.532)  /  2.525(1.048-6.080)  / | /  /  0.008*  /  0.039*  / | |
