# Supplementary material for: TGF-β1 promotes human breast cancer angiogenesis and malignant behavior by regulating endothelial-mesenchymal transition
Source: Front Oncol. 2022 Nov 16;12:1051148. doi: 10.3389/fonc.2022.1051148 (PMC9709251; doi:10.3389/fonc.2022.1051148)
Supplement: Supplementary file 4 [file DataSheet_1.zip › Supplementary Table 3.DOC]

**Table S3** Univariate and multivariate analysis of clinicopathologic factors for OS of IDC patients in our cohort

| Factor | OS | | | |  |
| --- | --- | --- | --- | --- | --- |
|  | univariate | | Multivariate | |  |
|  | HR (95%CI) | P value | HR (95%CI) | P value |  |
| Age (＜50 vs ≥50)  Tumor size  (＜2cm vs ≥2cm)  Lymphatic invasion  ( Yes vs no )  TNM (I vs II-III)  CD34 (Low vs High)  vWF (Low vs High) | 2.385(0.880-6.466)  35.72(0.512-2494)  6.206(2.039-18.89)  32.61(0.384-2773)  4.455(1.582-12.54)  3.642(1.049-12.64) | 0.088  0.099  0.001  0.124  0.005  0.042 | 3.459(1.204-9.942)  /  4.896(1.608-14.91)  /  4.269(1.482-12.30)  / | 0.021  /  0.005  /  0.007  / | |
