# Supplementary material for: TGF-β1 promotes human breast cancer angiogenesis and malignant behavior by regulating endothelial-mesenchymal transition
Source: Front Oncol. 2022 Nov 16;12:1051148. doi: 10.3389/fonc.2022.1051148 (PMC9709251; doi:10.3389/fonc.2022.1051148)
Supplement: Supplementary file 4 [file DataSheet_1.zip › Supplementary Table 4.DOC]

**Table S4** Chi-square test for the expression of vWF and CD34

|  | | vWF | | total |
| --- | --- | --- | --- | --- |
|  |  | low | high |  |
| CD34 | low | 32 | 20 | 52 |
|  | high | 4 | 30 | 34 |
| total | | 36 | 50 | 86 |

*P*<0.000
