# Supplementary material for: TGF-β1 promotes human breast cancer angiogenesis and malignant behavior by regulating endothelial-mesenchymal transition
Source: Front Oncol. 2022 Nov 16;12:1051148. doi: 10.3389/fonc.2022.1051148 (PMC9709251; doi:10.3389/fonc.2022.1051148)
Supplement: Supplementary file 4 [file DataSheet_1.zip › Supplementary Table 5.DOC]

**Table S5** Relationship between EndMT markers and MVD of IDC patients

|  |  | CD34 | | r | P  value | vWF | | r | P  value |
| --- | --- | --- | --- | --- | --- | --- | --- | --- | --- |
|  |  | LOW  (n=52) | HIGH  (n=34) |  |  | LOW  (n=36) | HIGH  (n=50) |  |  |
| α-SMA | LOW  HIGH | 36  16 | 12  22 | 0.334 | 0.002 | 25  11 | 23  27 | 0.233 | 0.031 |
| FSP-1 | LOW  HIGH | 27  25 | 10  24 | 0.222 | 0.039 | 23  13 | 14  36 | 0.358 | 0.001 |
| Vimentin | LOW  HIGH | 29  23 | 11  23 | 0.230 | 0.033 | 18  18 | 22  28 | 0.059 | 0.582 |
| Fibronectin | LOW  HIGH | 14  38 | 12  22 | -0.089 | 0.409 | 12  24 | 14  36 | 0.057 | 0.595 |
| VE-cadherin | LOW  HIGH | 31  21 | 14  20 | 0.181 | 0.094 | 19  17 | 26  24 | 0.038 | 0.943 |
