# Supplementary material for: TGF-β1 promotes human breast cancer angiogenesis and malignant behavior by regulating endothelial-mesenchymal transition
Source: Front Oncol. 2022 Nov 16;12:1051148. doi: 10.3389/fonc.2022.1051148 (PMC9709251; doi:10.3389/fonc.2022.1051148)
Supplement: Supplementary file 4 [file DataSheet_1.zip › Supplementary Table 6.DOC]

**Table S6** Primary antibodies for IHC

| Antibody | Concentration | Company |
| --- | --- | --- |
| CD34 | 1:400 | Abcam |
| vWF | 1:400 | Abcam |
| α-SMA | 1:600 | Cell Signaling Technology |
| FSP-1 | 1:400 | Abcam |
| Vimentin | 1:400 | Cell Signaling Technology |
| VE-Cadherin | 1:800 | Abcam |
| Fibronectin | 1:400 | Abcam |
| TGF-β1 | 1:500 | Abcam |
