# Supplementary material for: TGF-β1 promotes human breast cancer angiogenesis and malignant behavior by regulating endothelial-mesenchymal transition
Source: Front Oncol. 2022 Nov 16;12:1051148. doi: 10.3389/fonc.2022.1051148 (PMC9709251; doi:10.3389/fonc.2022.1051148)
Supplement: Supplementary file 4 [file DataSheet_1.zip › Supplementary Table 7.DOC]

**Table S7** Sequences of primers for qRT-PCR

| Gene | Forward primer | Reverse primer |
| --- | --- | --- |
| VE-Cadherin | TTTCACTGCAAACACACCTTGGA | GCTTTGCCCAGAGTGGGAAC |
| ACTA2/α-SMA | GCTTGTCAGGGCTTGTCCA | ACAGGAATTGAAGCGGAAGTC |
| VIM/Vimentin | AACCTGGCCGAGGACATCA | TCAAGGTCAAGACGTGCCAGA |
| FN1/Fibronectin | ACCCAGTCCCGAAGGCACTA | CCTGTACATCTAAAGGCATGAAGCA |
| Snail | GACCACTATGCCGCGCTCTT | TCGCTGTAGTTAGGCTTCCGATT |
| SNAI2/Slug | TTTCCAGACCCTGGTTGCTTC | CTCAGATTTGACCTGTCTGCAAATG |
| GAPDH  β-ACTIN | GCACCGTCAAGGCTGAGAAC  CGGGAAATCGTGCGTGAC | TGGTGAAGACGCCAGTGGA  CGCTCCAACCGACTGCT |
