# Supplementary material for: TGF-β1 promotes human breast cancer angiogenesis and malignant behavior by regulating endothelial-mesenchymal transition
Source: Front Oncol. 2022 Nov 16;12:1051148. doi: 10.3389/fonc.2022.1051148 (PMC9709251; doi:10.3389/fonc.2022.1051148)
Supplement: Supplementary file 4 [file DataSheet_1.zip › Supplementary Table 8.DOC]

**Table S8** Primary antibodies for western blot

| Antibody | Concentration | Company |
| --- | --- | --- |
| β-actin | 1:1000 | Cell Signaling Technology |
| GAPDH | 1:1000 | Cell Signaling Technology |
| vWF | 1:1000 | Abcam |
| α-SMA | 1:1000 | Cell Signaling Technology |
| FSP-1 | 1:1000 | Abcam |
| Vimentin | 1:1000 | Cell Signaling Technology |
| VE-Cadherin | 1:1000 | Abcam |
| Fibronectin | 1:800 | Abcam |
| Smad2/3 | 1:1000 | Cell Signaling Technology |
| p-Smad2/3 | 1:1000 | Cell Signaling Technology |
| Notch1 | 1:1000 | Abcam |
| Snail | 1:1000 | Santa Cruz |
| Slug | 1:1000 | Santa Cruz |
| TGF-β1 | 1:1000 | Abcam |
